# Expert opinion on metal chains and other indestructible objects as proper enrichment for intensively-farmed pigs

**DOI:** 10.1371/journal.pone.0212610

**Published:** 2019-02-22

**Authors:** Marc B. M. Bracke, Paul Koene

**Affiliations:** Wageningen Livestock Research, Wageningen University and Research, Wageningen, The Netherlands; US Geological Survey, UNITED STATES

## Abstract

EC Directive 2001/93 requires that all pigs have access to proper investigation and manipulation materials. Intensively farmed pigs in Europe are frequently provided with a short metal chain with or without an indestructible object attached to the chain. To date authorities are regarding this as proper enrichment, perhaps with (in)direct reference to the RICHPIG model as a justification. However, it has become increasingly clear that the chains do not provide proper enrichment, and that adding an indestructible object to the end of the chain may even reduce rather than improve pig welfare. To test this hypothesis an expert survey was conducted containing 26 more or less compound questions. On a scale from 0 to 10 experts specified their level of agreement with the hypothesis, the prevalence and welfare scores of nine indestructible enrichment materials. In total 36 experts, mostly pig-welfare scientists, responded (response rate: 39%). Indestructible objects are less prevalent in countries that provide straw (like Sweden and the UK) and outside the EU (US). They are more prevalent in the Netherlands, Belgium, France and Finland, while the prevalence seems to be low in Spain. Balls, wood and pipes were provided most frequently: hard wood especially in the UK (as specified in farm assurance); indestructible balls and pipes in Germany and the Netherlands. The experts’ score for agreement with the hypothesis was only 4.6 on average (scale 0–10; n = 25). Enrichment materials, ranked from high to low welfare score, were grouped in 5 significance levels as indicated by different superscripts based on Wilcoxon signed rank tests: Branched chains (5.1^a^), Chain on the floor (4.4^b^), Hard wood (3.7^bc^), Pipe (3.5^c^), Bare chain (3.3^c^), Short chain (3.1^d^), Small ball (2.8^d^), Big ball (2.5^d^), and Chain hanging too high (1.3^e^). Branched chains scored significantly better than all other indestructible materials and its welfare score (5.1 on average) was close to the pre-defined level of acceptability (5.5 on a scale from 0, worst, to 10, best). The welfare benefits of adding balls, pipes or hard wood to the metal chain were marginal, and well below what the experts considered acceptable enrichment. The branched-chains design, by contrast, appears to be the most viable alternative. It involves providing a longer chain, i.e. with the free end reaching to floor level, adding ‘branches’, i.e. several short chains ending at the nose height of the pigs, and providing more chains per pen (i.e. 1 branched chain per 5 pigs). Branched chains should be implemented widely and in the short term as a first step towards, and benchmark for, providing proper enrichment to intensively-farmed pigs.

## Introduction

In November 2015 the first author (MB) received an invitation to write a book chapter about the question what is proper enrichment for intensively farmed pigs [1]. This publication summarises about 15 years of working on this subject, including a number of noteworthy personal observations, several to date unpublished student reports, and about 15 peer-reviewed papers on enrichment and tail biting in conventionally-reared pigs. In particular, it describes how the RICHPIG model has been used to support decision making by the Dutch Ministry of Agriculture [2–5] to implement EC Directive 2001/93 requiring proper investigation and manipulation materials for pigs [6].

Based on the RICHPIG research the ministry decided that as of July 2007 Dutch pig farmers should provide more than just a metal chain [7]. In response pig farmers started to attach relatively hard hockey-type balls or polyethylene pipe to the commonly-provided short metal chain. Personal observations of the first author and results from student projects subsequently indicated concerns on the welfare benefits of these materials, and by the end of 2010 the first author had personally concluded that most probably pig welfare had failed to improve as intended by the new legislation, and that so-called branched chains could provide a feasible solution [8].

In particular, balls hanging on short chains on commercial farms were dry and collecting dust. When trying to bite the balls, the pigs failed to grab them, and the pigs interacted more with a short metal chain than with the same chain which had either a ball or a piece of pipe attached to it [1]. More importantly, perhaps, an ‘enriched’ chain, i.e. a much longer chain reaching to the floor and supplemented with short pieces of chain as ‘branches’, was much more appreciated by the pigs, even when kept on straw [1]. Unfortunately, no funds were available to empirically confirm, or refute, the hypothesis that the implementation of the EC Directive had reduced pig welfare. However, in the process of writing the book chapter (2015–2016) [1], it was feasible to do an expert survey to test the hypothesis and collect observations and expert views at an international level.

The objective of this survey, therefore, was to explore the views of international pig-welfare experts on the implications and perspectives of EC Directive 2001/93 requiring that all pigs have permanent access to proper investigation and manipulation materials, with a focus on the practice of providing indestructible materials to intensively-farmed weaned and growing/fattening pigs. In particular, a specific aim was to test the hypothesis whether welfare had been reduced by adding an indestructible object to (the end of) the short metal chain.

## Methods

On February 14 2016 a questionnaire (see supporting information S1 Survey) was sent to 13 experts by email. In addition, 59 experts received a request to participate, without enclosing the survey itself. Respondents were asked to provide names of other experts (Question Q17 and Q18 in S1 Survey). This resulted in a further 21 contacted experts. Hence, in total 93 experts were contacted. These were mostly senior pig-welfare scientists and scientists with relevant expertise on pig enrichment in intensive farming (based on scientific publications and/or current research projects).

The questionnaire consisted of a table with 26 rows containing more or less compound questions, and 4 columns for up to 4 answer fields per question (S1 Survey). It was introduced as follows:

“Presently, I’m reviewing enrichment materials for intensively-farmed pigs. Below you find a brief questionnaire for experts, i.e. scientists, veterinarians, farm advisors with an academic background and extensive practical experience.

I need help because this is an important as well as problematic issue. My focus is on chains and indestructible objects for esp. weaners and growing/fattening pigs kept in conventional pig farms (i.e. excluding pigs in specific welfare schemes or organic farming). Indestructible objects hanging on a chain are widely used in Europe esp. following the implementation of EC Directive 2001/93 stating that all pigs must have permanent access to a sufficient quantity of material to enable proper investigation and manipulation activities. My own research involving reviewing, modelling and on-farm work on this subject (see eg "Bracke enrichment pigs" in Google Scholar) may have been at the basis of the political decision in the EU that chains alone were no longer considered to be sufficient [7, 9, 10]. This led many farmers in (mainly N-W) Europe to attach to the chain a piece of rather indestructible plastic pipe, ball or (hard)wood. Unfortunately, I'm also (next to) convinced that as a general rule this implementation may in fact have reduced pig welfare. Therefore, I need your help to either confirm or refute my hypothesis, as well as to specify what would be adequate enrichment.”

Received responses were collected in an Excel datasheet (see S1 Dataset). In some cases a short clarification was requested when an answer was not sufficiently clear. Experts were not required to complete the whole questionnaire.

The experts were asked to give several scores on a scale from 0 to 10. Two agreement scores expressed agreement of the expert him/herself (Q2a) and the general perception in practice (Q2b) with the hypothesis on a scale from 0, totally disagree, to 10, totally agree. In addition, up to 14 welfare scores could be given. Welfare scores were expressed on a scale from 0 to 10, i.e. compared to a pen without any enrichment and compared to the best possible enrichment respectively. On the welfare scale, a score of 5.5 was specified as the cut-off point for what the respondent would consider adequate/sufficient enrichment for pigs.

Exploratory data summaries (e.g. average and standard error, S.E.) were calculated using Microsoft Excel. Statistical analyses were performed using IBM SPSS version 22.

For the main analysis, nonparametric tests were used. To identify the presence of differences between dependent variables (i.e. predefined enrichment materials in Q8a, Q9a-d and Q10a-d) a multivariate Friedman test was used for ordinal welfare scores, followed by pairwise comparisons using a related-samples Wilcoxon signed rank test when the Friedman test was significant. The adjusted P values are presented. Significant differences (p<0.05) between enrichment materials were used to identify groups of materials that differed significantly. In order to further examine the effect of geographical region (based on answers to Q1) and gender (based on the experts’ names and public profiles), several transformations of the welfare scores were performed. The log-transformation ((log(welfare score+1)) showed a strong improvement in data normality. A subsequent REML analysis was done with fixed factors ‘Enrichment material’, Region and Gender. The factor Region tentatively classified experts in regions broadly sharing similar types of enrichment (as indicated in the answers of Q4-7). The final REML model contains all significant factors and interactions underlying the welfare scores given to the predefined enrichment materials (Q8a, Q9a-d, Q10a-d). Cronbach’s alpha was calculated to examine the level of agreement between the experts in providing welfare scores. Presented means are based on original (untransformed) data.

## Results

Of 93 experts contacted, 36 experts responded of which 4 experts responded as 2 pairs (overall response rate: 39%). Three experts responded without giving any numerical score. Below, the results are shown per question (Q). Responses to questions most relevant to the testing of the hypothesis and welfare assessment are presented here. A summary of responses to the remaining questions is presented in supporting information S1 Answers (to Q3 and Q11-25).

**Q1**. **Which country are you knowledgeable about?**

Out of 36 respondents 6 were not from Europe (US: 4; Canada: 1, New Zealand: 1). Most represented were the UK and the Netherlands (7 and 5 respondents respectively). Belgium, Spain, France, Italy and Sweden had 3 respondents each. Finland 2 and 1 from Switzerland and Germany. Notably no Danish respondent was included, but 2 experts indicated representing expertise about Denmark. The average knowledge score (Q16a, scale 0–10) was 7.6 (S.E.: 0.3, n = 27).

**Q2a and Q2b**. **Do you agree with the hypothesis that generally the welfare of intensively-farmed pigs has been reduced by adding an indestructible object to (the end of) the metal chain? (Q2a). Is this the general perception in practice (i.e. among farmers, vets and extension workers)? (Q2b)**.

Expressed on a scale from 0 (totally disagree) to 10 (totally agree) the average expert agreement score was 4.6 (S.E.: 0.5; n = 25) (see Table 1). The average agreement score for the general perception in practice was: 3.4 (S.E.: 0.6; n = 18).

**Table 1:** Numerical results from the questionnaire.

Q.No	Question	Average	Count	S.E	Min	Max
2a	Agreement that indestructible objects have reduced pig welfare	4.60	25	0.50	0	8
2b	Is this generally known in practice?	3.44	18	0.60	0	9
4a	Prevalence (P) of a metal chain	43.13	8	12.21	5	90
4b	From	15.71	7	8.05	0	60
4c	To	31.25	8	7.43	5	70
5a	P of indestructible objects	41.00	5	16.46	5	90
5b	From	26.38	8	8.80	0	60
5c	To	36.30	10	11.07	3	90
7a	P of the main indestructible objects	32.15	11	8.95	0	70
7b	P of the next most freq. indestr. obj.	14.80	7	5.06	1.6	40
7c	P of third most freq. indestr. obj.	7.15	4	3.25	1.6	15
8d	WS of a bare chain	3.32	22	0.38	1	6.5
9a	WS of a pipe	3.52	27	0.38	0.5	7
9b	WS of a small ball	2.80	27	0.33	0.5	6
9c	WS of a big ball	2.52	26	0.34	0	6
9d	WS of (hard) wood	3.74	27	0.46	0	8
10a	WS of a short chain (1 per 15 pigs at nose height)	3.08	28	0.32	0.5	6
10b	WS of a chain hanging too high	1.34	28	0.28	0	5.5
10c	WS of a chain on the floor	4.36	28	0.40	1	7.5
10d	WS of branched chains (1 per 5 pigs)	5.07	28	0.43	1	8
11	WS under optimised conditions	7.13	8	0.57	4.5	9
12	WS under inadequate conditions	4.30	5	1.43	0	9
16a	Knowledge score	7.56	27	0.28	5	10

For more detailed formulation of the questions see the text and the full questionnaire in supporting information S1 Survey using the question number (Q.No) as key. P: Prevalence; WS: Welfare score; S.E.: Standard error.

**Q3**. **Do you have personal observations or referenced sources of information either supporting (3a) or refuting (3b) my hypothesis?**

3a. 12 affirmative and 11 negative answers were received. 3b: 5 affirmative and 13 negative answers. In total 21 publications were suggested [11–33], and 23 clarifications. The latter were mostly personal observations. All suggestions, including the ‘refutations’ were compatible with the hypothesis (see S1 Answers for more details and expert citations).

**Q4 and Q5**. **What is the prevalence of metal chains without (Q4) and with (Q5) indestructible objects attached to the chain in conventional pig pens in your country?**

The overall average prevalence of the chain with and without indestructible objects attached to the chain was: 41% (S.E.: 16.5; n = 5) and 43.1% (S.E.: 12.2; n = 8) respectively.

Supporting information S1 Table gives a breakdown of stated prevalence by geographical region. It shows considerable variation in the use of metal chains and indestructible objects. It is low in countries that provide straw (like Sweden and the UK) and outside the EU (US). Metal chains, with or without indestructible objects attached to the chain, seem to be prevalent in the Netherlands, Belgium, France and Finland, while the prevalence seems to be low in Spain (S1 Table).

**Q6 and 7**. **What are the main indestructible objects attached to chains in your country, and what is their prevalence?**

Balls, (hard) wood and plastic pipes were mentioned most frequently. The UK often uses hard wood as specified in farm assurance for pens without straw. (Hard) balls and (hard) pipes are frequently used in Germany and the Netherlands.

Few hard data seem to be available. A Dutch dataset indicated that in 2011 in 27.5% of conventional pens only a chain was provided, and in 19.5% of pens a chain with a ball was present (in a total of 841 pens observed at 47 farms; see S1 Fig). Our impression is that compared to 2011 there is tendency for fewer bare chains, fewer balls and more plastic pipes being provided on conventional Dutch pig farms at present.

**Q8**. **Give a welfare score for the materials specified in the previous questions, including the metal chain (without attachments)**

Since the objects specified largely overlapped with the pre-defined objects in the next question, the answers to this question were not analysed in detail.

Average welfare scores for whatever the experts specified as first, second and third most prevalent indestructible object in their country (Q8a, 8b and 8c) ranged between 3.82 and 3.94. The bare metal chain (without attached object, Q8d) had an average welfare score of 3.3 (S.E.: 0.4; n = 22).

**Q9 and Q10. What welfare scores would you give to the following indestructible objects attached to the end of the chain at nose height of the pigs: A: Pipe; B: Hockey-size ball; C: Big ball (size: small football); D: (Hard) wood? (Q9). Q10: What scores would you give to the following types of chain**:

**10a**. **1 chain for up to 15 pigs with the chain ending at nose height of the smallest pigs entering the unit (labelled in the text as ‘Short chain’).**

**10b: as Q10a but hanging too high for a large majority of the smallest pigs (labelled as ‘Chain hanging too high’)**.

**10c: as Q10a but reaching to floor level (solid floor) such that pigs can also 'root' the end of the chain and can manipulate the chain while lying (labelled as ‘Chain on the floor’)**.

**10d: as Q10a but now 1 chain for every 5 pigs (such that more pigs can play (i.e. use the object) at the same time), and chains ending at various heights, including both at nose height and floor level (labelled as ‘Branched chains’)**.

**Fig 1:**
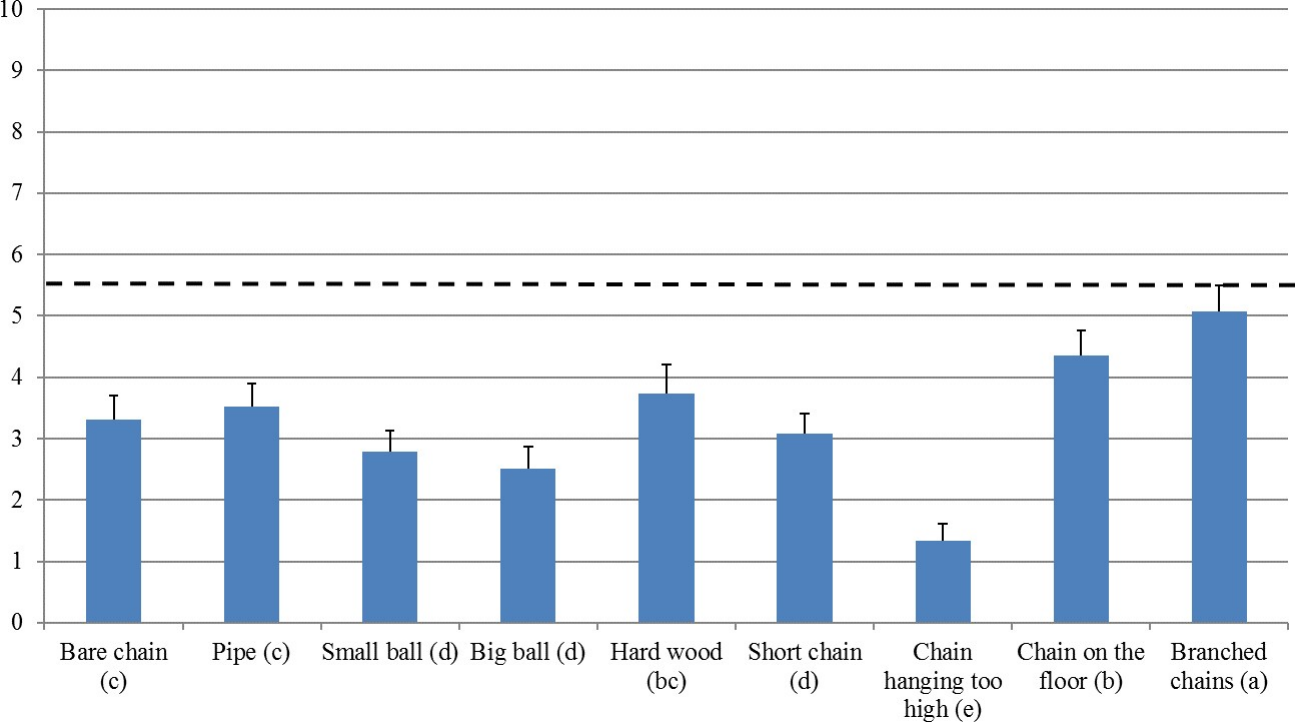
Average welfare scores and standard errors for the various enrichment materials as specified in questions Q8d (Bare chain), Q9a-9d (Pipe, Small ball, Big Ball, Hard wood) and Q10a-10d (Short chain (1 for up to 15 pigs), Too high, On the floor, Branched chains). N = 22–28 (see Table 1). Characters behind material labels between brackets are superscripts indicating significant differences as indicated by pairwise Wilcoxon signed rank tests. The predefined cut-off line between acceptable and unacceptable enrichment is 5.5 on a scale from 0, worst, to 10, best.

The Friedman test was highly significant (p<0.000; Chi square 90.5, df: 8). Enrichment materials, ranked from high to low welfare score, were grouped in 5 significance levels as indicated by different superscripts based on Wilcoxon signed rank tests: Branched chains (5.1^a^), Chain on the floor (4.4^b^), Hard wood (3.7^bc^), Pipe (3.5^c^), Bare chain (3.3^c^), Short chain (3.1^d^), Small ball (2.8^d^), Big ball (2.5^d^), and Chain hanging too high (1.3^e^) (see Fig 1, and Tables 1 and 2). The first significance level consisted of Branched chains only. This enrichment material was significantly better than all other materials. The lowest level was Chain hanging too high. It was worse than all other materials. Only one material, Hard wood, was member of both the second level (together with Chain on the floor) and the third level (with Pipe and Bare chain). In addition, two statistical trends were identified. Within the second level, Chain on the floor tended to have a higher welfare score than Hard wood (p = 0.062), and within the fourth level Short chain tended to have a higher score than Big ball (p = 0.068; Table 2).

**Table 2:** Results of pairwise Wilcoxon signed rank tests (p-values) for the various materials as specified in questions Q9a-9d (Pipe, Small ball, Big ball, Hard wood), 8d (Bare chain) and Q10a-10d (Short chain, Chain hanging too high, Chain on the floor, Branched chains), sorted by average score and Friedman mean rank.

	Branched chains	Chain on the floor	Hard wood	Pipe	Bare chain	Short chain	Small ball	Big ball	Chain hanging too high	Mean Rank	Level 1	Level 2	Level 3	Level 4	Level 5
**Branched chains**		0.000	0.001	0.000	0.000	0.000	0.000	0.000	0.000	8.21	a				
**Chain on the floor**	0.000		0.062	0.011	0.004	0.000	0.000	0.000	0.000	6.84		b*			
**Hard wood**	0.001	0.062		0.364	0.623	0.003	0.007	0.003	0.000	5.61		b#	C		
**Pipe**	0.000	0.011	0.364		0.697	0.037	0.024	0.009	0.000	5.53			C		
**Bare chain**	0.000	0.004	0.623	0.697		0.017	0.017	0.013	0.000	5.42			C		
**Short chain**	0.000	0.000	0.003	0.037	0.017		0.455	0.068	0.000	4.26				d*	
**Small ball**	0.000	0.000	0.007	0.024	0.017	0.455		0.168	0.000	4.18				d*#	
**Big ball**	0.000	0.000	0.003	0.009	0.013	0.068	0.168		0.000	3.71				d#	
**Chain hanging too high**	0.000	0.000	0.000	0.000	0.000	0.000	0.000	0.000		1.24					e
**Count (n)**	28	28	27	27	22	28	27	26	28						
**Count > 5.5**	14	9	6	6	4	3	2	1	0						
**Average**	5.07	4.36	3.74	3.52	3.32	3.08	2.80	2.52	1.34						

Count (n) gives the number of respondents providing a welfare score (scale 0 to 10). Count > 5.5 are numbers of respondents giving a score that is higher than 5.5, which was specified as the cut-off point for acceptability. Cells marked green represent statistically significant pairwise comparisons (p<0.05). Light green: trend (p<0.1); Yellow: not significant. Levels 1–5 show groups of enrichment materials, sorted from high to low, where different characters (a-e) indicate levels of statistical significance, and where different symbols (*, #) indicate statistical trends (so d*, Short chain, tends to differ from d#, Big ball (p = 0.068), but not from d*#, Small ball).

When comparing the level of welfare scoring between experts, it was noted that most experts generally gave very low scores, while others gave considerably higher scores. Out of 28 experts who provided welfare scores, 13 experts had an overall average welfare score below 3.0 (across different chains and indestructible objects as specified in Q8d, Q9 and Q10). Eight experts even scored on average below 2.0 (4 were men and 4 women; 4 from the UK, 1 from Canada, 1 from the Netherlands, 1 from the US and 1 from Finland). By contrast, 4 experts had averages above 5.0 (1 men, 3 women; 3 from Italy, 1 from the Netherlands). The difference between experts in position on the welfare scale was confirmed by highly significant Spearman rank correlations between enrichment materials (all Spearman Rho above 0.61 and all P<0.001), indicating that each expert had a fairly consistent rank position in the rank order of expert welfare-scores per enrichment material.

It should be noted that 5 experts provided scores requiring clarification, e.g. when a lower welfare score was given to more chains being provided in the pen. These experts generally confirmed having misunderstood the question and then modified their scores accordingly.

The Cronbach’s alpha was very high (0.96), indicating that the experts agreed in scoring (especially in ranking) the different enrichment materials.

In the final REML model (S2 Table) main factors and most interactions were significant. The interaction between Gender * Region was not significant and therefore removed. This was also true for the factor ‘Agreement score with the hypothesis’ (Q2a).

The REML analysis indicated that women provided substantially higher (!) welfare scores than men (back-transformed overall means were 3.3 and 2.0 respectively; p = 0.02), and that there was a main effect of Region (p = 0.01). Somewhat surprisingly again, experts from non-EU countries, where little or no enrichment material is provided (US and Canada), and from countries in upper-NW Europe, where straw provision was common (UK, Sweden) and/or tail docking was banned (Finland), gave significantly lower welfare scores (back-transformed means: 1.9 and 1.7 respectively) than experts from EU countries where no enrichment was provided or where a chain was considered sufficient (Italy, Spain, France, Belgium; mean: 4.3). Intermediate scores (not differing significantly from the previously-mentioned three regions: Non-EU, upper-NW and South-middle Europe) were given by Dutch and German experts (NW continental Europe), where a bare chain alone was no longer considered sufficient since the EC Directive had been implemented (mean: 2.8).

Finally, the REML analysis showed a similar classification in five levels as reported for the Friedman test. However, most interestingly, Hard wood now scored significantly lower than Chain on the floor (p = 0.001, thus moving from intermediate between the 2^nd^ and 3^rd^ level to the 3^rd^ level). In addition, Short chain moved up from the 4^th^ level to being intermediate between the 3^rd^ and 4^th^ level, thus as before not being different from Small ball and Big ball in level 4 (p values 0.2 and 0.1 respectively), but now also no longer being significantly different from Hard wood, Pipe and Bare chain in level 3 (p values 0.2, 0.96 and 0.06 respectively; so there was still a trend difference with Bare chain).

**Q11-25**. The answers to the remaining questions 11–25 are presented in supporting information S1 Answers.

## Discussion

Below the answers to the questions (Q) in the survey are discussed in view of the objective of this paper, i.e. to formulate an international expert-opinion on the effects of providing indestructible materials on the welfare of intensively-farmed weaned and growing/fattening pigs. The results are also discussed in relation to previous research on this subject as presented and discussed in the aforementioned book chapter [1].

**Q1**. Most respondents were European (84%), of which most were NW European (61%). Countries in NW Europe, including the UK and the Netherlands, generally have a longer history of societal debate and government-funded research on farm-animal welfare. Furthermore, the survey concerned the implementation of EC Directive 2001/93 requiring proper enrichment for intensively farmed pigs [6]. In particular, we examined the level of expert consensus on the author’s (MB’s) personal observations and hypothesis. This may partly explain the relative overrepresentation of Dutch respondents (14%).

**Q2 and 3**. The main objective was to examine the hypothesis that as a general rule the welfare of intensively-farmed pigs in Europe had been reduced since indestructible objects had been added to (the end of) the metal chains as a way to implement the Directive. The hypothesis was presented for one-sided evaluation (i.e. the experts were not asked more neutrally whether welfare had reduced, stayed the same or had improved). The reason for this one-sided ‘testing’ was that the first author’s previous observations had repeatedly indicated that the pigs manipulated the new materials less frequently and sometimes appeared frustrated by them when trying to interact with them [1], and this was thought to justify a one-sided ‘test’. However, with an average agreement score of 4.6 (on a scale from 0 to 10) and an even lower score for the general perception in practice (3.4), the experts did not confirm the hypothesis, i.e. they were only partially aware of the problem themselves and it was generally even less well known in the pig industry. The ambivalence was also reflected in the welfare scores assigned to different enrichment materials (discussed in more detail below). A possible explanation may relate to the variable prevalence of chains and other indestructible objects provided to pigs across Europe (Q4-7).

The agreement score with the hypothesis also did not correlate with the welfare scores given to any of the enrichment materials (Q8d, Q9-10). In a tentative principal component analysis, the agreement score came out as one of two main explanatory variables. The other main factor was comprised of the welfare scores for the different materials, which were all highly correlated (see below).

**Q4-7**. Relatively few experts specified the prevalence of enrichment materials. This may relate to the fact that by far most respondents were scientists, rather than vets or farm advisors (Q16).

The obtained responses, however, suggest that considerable differences exist between countries and geographical regions. Outside the EU most often no enrichment materials are being provided to intensively-farmed pigs. In Europe, Sweden requires straw, and about 50% of the growing pigs in the UK are kept on straw, while most other pigs in the UK are provided with hard wood on a chain as this is required by the prevalent welfare scheme. Countries like the Netherlands and Germany mostly provide plastic pipes or balls on the chain. In France and Belgium chains without destructible objects appear to be prevalent. The Belgian government implemented the EU Directive with a regulation that as of April 1 2014 a bare chain was sufficient for weaned pigs and for growing/fattening pigs, provided it was hanging at least 10 cm from the pen wall (and that a moveable plastic tube was sufficient for farrowing sows [34]). Tubes on bars of farrowing pens and feeding stalls were prevalent for sows in the Netherlands too, but to the authors’ knowledge no scientific evidence is available to support this practice.

**Q8-10**. The experts provided welfare scores (WS, scale 0–10) for 9 predefined, indestructible enrichment-materials for intensively-farmed pigs. The results were remarkable in at least four respects. Firstly, experts largely agreed on ranking, but they did not agree on what is acceptable enrichment. Secondly, two seemingly similar characterisations of the most prevalent metal chain (Bare chain in Q8d and Short chain in Q10a) differed significantly. Thirdly, the welfare scores for the enrichment materials only partially supported the hypothesis that adding indestructible objects to the short/bare metal chain reduced pig welfare. Fourthly, Branched chains provide a substantial welfare improvement and it constitutes almost acceptable enrichment.

Sorted by average welfare score the 9 materials were grouped into five welfare levels as follows: Branched chains (5.1^a^), Chain on the floor (4.4^b^), Hard wood (3.7^bc^), Pipe (3.5^c^), Bare chain (3.3^c^), Short chain (3.1^d^), Small ball (2.8^d^), Big ball (2.5^d^), and Chain hanging too high (1.3^e^), where different superscripts indicate significant differences (p<0.05).

Experts appeared to agree much more on the rank order of the materials than on their level of acceptability as proper enrichment. Some experts gave much higher scores than others. Eight experts gave very low scores (i.e. their overall average welfare score for all materials was below 2.0). By contrast, 4 experts had average values above 5.0. Three experts even scored above 5.5, which was the pre-defined cut-off point for acceptability. In other words, some experts indicated that all of these indestructible materials provided clearly inadequate, i.e. improper, enrichment, while others held that most materials were acceptable. In providing welfare scores experts may have related to what is considered ‘normal’ for conventional pig production in their region, and they may have selected different criteria to define acceptability. Experts guided by the need to reduce tail biting may have given low scores, while experts thinking more about production, health or stress-physiology may not have seen much of a problem with any of these materials [2].

The noteworthy consensus among experts on the ranking of the materials is emphasised by the fact that both the best and the worse material, Branched chains and Chain hanging too high respectively, differed significantly from all other materials. Another remarkable finding concerns the significant difference (p = 0.02) between Bare chain (Q8d) and Short chain (Q10a). These materials had very similar average welfare scores (3.3 and 3.1 respectively), and this difference, while being significant in the Friedman test, was reduced to a trend in the tentative REML analysis, p = 0.06). Out of 21 experts providing a score for both items, 14 experts gave exactly the same score, 7 experts gave a lower score to Short chain than to Bare chain, and no expert gave a higher score to Short chain. Short chain was explicitly specified in Q10a of the questionnaire as “1 chain for up to 15 pigs with the chain ending at nose height of the smallest pigs entering the unit”. This was more or less common practice in the Netherlands until 2007 when the EC Directive was implemented. Bare chain in Q8d was defined as ‘chain without any attached objects’. For the authors, and probably for most of the 14 experts giving tied scores, these formulations are referring to the same and most well-known practice in intensive pig farming in Europe. So, if it wasn’t a random difference, why then did 1/3 of the respondents give a lower score to Short chain? Perhaps the experts had slightly different chains in mind (e.g. Bare chain in Q8d could have been perceived to be a bit longer or a bit more prevalent, e.g. one chain for every 10 pigs rather than one in 15 as specified for Short chain). Another explanation may be a slight change of context (direct comparison). Bare chain in Q8d served as a comparison to the indestructible materials specified by the expert as most prevalent in his/her region in Q6. Short chain in Q10 immediately followed scoring of the materials in Q9 (Pipe, Small ball, Big ball and Hard wood), and in Q10 Short chain was compared to other types of chain (Chain hanging too high, Chain on the floor and Branched chains). The materials in Q9, especially Pipe and Small ball, were formulated in accordance with the implementation of the EC Directive in the Netherlands. The main objective of the comparison in Q10 was to explore expert opinion regarding the first author’s (MB’s) experience that Branched chains are a considerable improvement for pig welfare compared to Short/Bare chain (and various other types of chain like Chain on the floor [1]). Perhaps the experts (i.e. the 7 experts giving a lower score to Short chain than to Bare chain) reduced the welfare score assigned to Short chain due to the higher welfare scores attributed to Chain on the floor and Branched chains, which were both significantly better for pig welfare than both Short chain and Bare chain, and which generally appeared to be limited at the upper end by 5.5 as the cut-off point for acceptability.

By far the two most important findings related to the welfare scores elicited in this survey, however, concern the limited confirmation of the main hypothesis and the general support for the proposition that Branched chains may substantially improve the welfare of intensively-farmed pigs.

The main hypothesis was that pig welfare had reduced across Europe due to indestructible objects attached to the end of the prevalent (Bare and Short) metal chain following the implementation of EC Directive 2001/93. The hypothesis was rejected in that Hard wood (3.7^bc^) and Pipe (3.5^c^) had significantly higher welfare scores than Short chain (3.1^d^) (but not significantly higher than Bare chain (3.3^c^)), and in that Small ball (2.8^d^) did not differ significantly from Bare chain. (Note, also, however, that the significant difference between Hard wood and Pipe on the one hand and Short chain on the other no longer applied in the REML analysis.) In one respect, experts’ welfare scores did support the hypothesis, namely in that Small ball (2.8^d^), which is prevalent especially in the Netherlands and Germany, received a significantly lower welfare score than Bare chain (3.3^c^). However, Small ball (2.8^d^) was not significantly lower than Short chain (3.1^d^), though the welfare score was lower on average (2.8 versus 3.1). With this limited support for the hypothesis, the proposition, that the practice of attaching an indestructible object to the short metal chain significantly reduced pig welfare, should be regarded as rejected in this expert survey. The proposition is based on unpublished empirical observations of the first author on different farms and in different categories of pig, all indicating that pigs use the end of a short/bare chain more than the same chain with an indestructible object attached to the end of it [1]. It also relates to the classical study by Feddes and Fraser [14] showing that both cotton cord and a rubber strip were chewed much less when presented in a loop (i.e. without exposed end) compared to being presented as a straight end (cotton: 2 min/day versus 30 min/day; rubber strip: 1 min/day vs 12 min/day). Both Hard wood and Small ball do not provide a ‘free end’ to the pigs, whereas a short, hanging chain does, to some extent. The pig can bite it and move it around in the oral cavity (as the chain links can be moved relative to each other). Pipe may also be perceived as having a sufficiently small diameter to be regarded as a biteable ‘free end’. However, a student showed that dry sows manipulated (the end of) a short chain five times more frequently than exactly the same chain end covered by a small piece (10 cm) of tough polyethylene piping, as commonly used on commercial pig farms in the Netherlands [35]. Several studies report somewhat more positive results of pipe presented as longer rubber pipes, either hanging [15, 36] or on the floor [20, 37, 38]. However, these designs appear to be less representative of what is provided on commercial farms (where the amount of material used is less and where what remains hanging in the pens is nearly indestructible).

In formulating the hypothesis, the first author was expecting that what he had seen in the Netherlands would be more or less representative of other European countries as well. This did not appear to be the case. The implementation of the EC Directive on pig enrichment varied greatly across Europe (see the discussion of answers to Q4-7 above). In fact, the hypothesis may only apply to the Netherlands and Germany, where Pipe and Small Ball are common. The hypothesis does not appear to apply to countries like Sweden and the UK where many farmers already provide straw, or to countries that have already banned tail docking and thus stimulated the provision of better enrichment materials (e.g. Finland). Finally, the hypothesis does not apply to most pig farms in the UK that do not provide straw, because these provide Hard wood as a requirement for welfare assurance. If so, these farms could formally be disqualified, because the questionnaire explicitly excluded pigs in welfare schemes. Many pigs in the Netherlands are raised under the Better Life welfare scheme, and those farms were excluded, because they normally provide some destructible material. However, since Hard wood qualifies the description in Q9d and would qualify under Dutch regulations for conventional pig farming, it should not be disqualified conceptually. However, wood may be so hard, that growing/fattening pigs only manage to ‘scratch’ the surface of the wood, and that it is hardly possible to cut the wood using a handsaw. Such hardwood logs (about 10 cm in diameter, 40 cm long, lying on the floor attached to a short chain) had no value in reducing tail biting problems and appeared to have little if any added value for manipulation compared to the free end of a chain (MB, pers. obs.). In fact, even soft wood may sometimes qualify as ‘Hard wood’ as defined in Q9d, as it was specified as ‘(Hard)wood …more or less indestructible/very long lasting”. For example, a wooden log may be too big to bite (e.g. having a diameter of 20–30 cm) and/or the log may be suspended on a free hanging chain such that it moves out of the way when pigs try to bite the log. If so, the suspended wood may actually swing back, like a small ball on a chain, and hit the pig. Such soft wood and small balls mainly induce interactions indicative of frustration and may well have a negative overall enrichment value. This is in line with Nannoni et al. [32] who compared a chain hanging in the middle of the pen with a log of poplar wood (which is soft wood, trial 1) and with an edible block (made of feed, alfalfa meal, sugar beet molasses, and minerals; trial 2). Both the wood and the edible block were cylindrical: 10 cm diameter * 25 cm, free to rotate and bite, and hanging horizontally in a metal frame on the pen wall, 10 cm above the withers of the weaned piglets. They found no significant difference in object manipulation, but in each of these two comparisons the chain was manipulated on average (a bit) *more* than the poplar, i.e. soft wood log, and also a bit more than the edible block. This indicates that even the new Commission Recommendation (EU) 2016/336 [29], which requires that enrichment materials should be *edible*, *chewable*, *investigable and manipulable*, may ‘incidentally’ lead to a reduction in pig welfare. It also indicates that further optimisation of the metal chain may lead to more welfare improvement at a lower cost, also according to this expert survey, compared to the most economical ‘compliance’ with the recommendation (which is not legally required, and therefore probably also difficult to implement given the economic constraints on intensive pig farming).

Without suggesting that other materials are always worse (e.g. see Telkänranta et al. [15] and S1 Answers on the use of birch wood) and without implying that the experts may have been mistaken, it is clear that (public) perceptions may differ from what really matters to pigs. Wood is a natural material. A chain is not. A chain may, perhaps subconsciously, be associated with imprisonment and slavery. By contrast, we may associate large and small balls with playing games like soccer and hockey, but pigs, of course, do not have any such associations. Furthermore, a bare chain is a single material. A chain with an object attached to it, may, wrongly, suggest compound/multiple enrichment, namely derived from both the chain and the object. However, the remaining enrichment value of the hanging chain itself may in fact be reduced to (almost) zero by adding a ball, pipe or wood to the end of the chain. The less manipulable object may replace the manipulable end of the chain, and that, more in particular, was the point of the hypothesis. In the Netherlands, the immediate and general response of pig farmers was to replace the end of the short chain with Small ball or Pipe. Especially the balls were observed collecting dust, and vets complained the balls hit them when they vaccinated the pigs. The balls also hit the pigs when they tried to bite them. These and other observations, described in more detail in the book chapter [1], as well as the failure of this expert survey to confirm the hypothesis, would therefore certainly merit further empirical work. Multiple student projects could be formulated making an inventory of the current practices on commercial farms, and comparing these practices to the free end of a metal chain and to the Branched-chains design (1 branched chain per 5 pigs), acting as benchmarks.

Although the hypothesis was mostly rejected, the expert survey did indicate that the prevalent indestructible objects (Pipe, Small Ball and Hard wood) actually failed to benefit the pigs as intended by the EC Directive. Small ball had a lower average welfare score (2.8^d^), and, while Hard wood (3.7^bc^) and Pipe (3.5^c^) scored significantly higher than Short chain (3.1^d^), they did *not* score significantly higher than Bare chain (3.3^c^), which was a highly similar material to Short chain (see above). Furthermore, the welfare scores of these most prevalent materials had a narrow range (2.8–3.7) that remained well below the cut-off point of acceptability (5.5). This finding confirms earlier reports and expert scoring [3, 25, 26]. A final point of concern about the state of pig welfare in Europe regarding enrichment, and confirmed in this study, was the very low score for Chain hanging too high (1.3^e^). It was defined as “hanging too high for a large majority of the smallest pigs”. Pigs grow rapidly and maximising cost-efficiency may motivate farmers to hang the (more or less destructible) materials as high as possible, so as to make them more ‘durable’. This may generate a concern for pig welfare on commercial farms.

The last remarkable and most important finding related to the welfare scoring in this study was that Branched chains provide superior and almost acceptable enrichment. Branched chains was defined as 1 chain for every 5 pigs (such that more pigs can manipulate it at the same time), and chains ending at various heights, including both at nose height and at floor level. Reaching to floor level (on a solid floor) implies that pigs can also 'root' the end of the chain and can manipulate the chain while lying (see also http://farewelldock.eu/branched-chains-enrichment-pigs-technical-description-pictures/).

Branched chains is a further design improvement compared to the Chain on the floor, which was the second best material, scoring significantly better than all (7) other materials. This is in line with the author’s impression (and empirical observations) that making the short chain longer and longer, further improves its enrichment quality (unless the end gets stuck between the slats) [1]. It is also in line with a study where a longer chain (extending from the ceiling to 10 cm above the floor, so not extending to floor level) was shown to substantially reduce ear lesions in weaned piglets that had limited access to water [39].

Branched chains was deliberately formulated in plural to emphasise that it implies providing several branched chains in a pig pen, and that each branched chain has multiple chain ends that can be manipulated by the pigs at different heights and positions. Therefore, the Branched chains differed from the other chains in presenting multiple chain ends (branches), as well as in being available at a higher ‘concentration’ (1 chain per 5 pigs, instead of up to 15 pigs). Since this was a compound improvement, the current survey does not allow a specification of how much each aspect of the Branched-chains design contributed to its overall welfare improvement.

As a group the experts considered Branched chains to be significantly better than each of the 8 other materials. In addition, with an average welfare score of 5.1 (S.E.: 0.4; n = 28) it was on average only 0.4 welfare points below the acceptability threshold (5.5). This difference is very small, and it is conceivable that adding otherwise inferior indestructible objects to the Branched chains may be sufficient, according to the experts. However, since this was not included in the survey, the suggestion remains speculative.

This raises the question what may be the role of Branched chains in providing proper enrichment materials for manipulation. It failed to actually reach the cut-off point. Experts also strongly recommended providing destructible materials to improve the current practices (see Q13 in S1 Answers). However, concern is justified that available destructible materials may not prove to be feasible in current intensive pig-farming practice in Europe, unless more rigidly defined in legislation and actively enforced [40]. Recently, the European Commission (EC) drafted guidelines recommending the use of destructible (edible and chewable) materials [28, 29]. However, since the EC Directive requires that proper enrichment must be permanently available to all pigs, providing destructible materials permanently is probably very difficult given the current economic constraints on intensive pig production. Since a feasible alternative is lacking, the widespread implementation of Branched chains in intensive pig farming, not only in Europe, but across the world, is recommended. It provides a unique opportunity to considerably improve pig welfare at the lowest possible cost and risk (in terms of labour, blocked manure systems, pig health and environmental concerns [1, 41]). In the former publication the author provides more details on how to optimise the Branched-chains design further, e.g. concerning the type of chain to be used and optimised feasibility (eg last 5–10 links made of stainless steel anchor chains, 7 mm for growing-fattening pigs, 5–6 mm for weaners [1]). The design may be tested and improved further using the principle of Intelligent Natural Design [1]. The author-version of this publication and technical specification is available via http://farewelldock.eu/chain-as-enrichment-with-supplement/.

**Q11-15**. Experts mentioned a range of familiar material properties [4, 5] specifying how enrichment materials could be optimised (Q11, S1 Answers) and what is relevant to avoid suboptimal use (Q12). Experts also suggested a range of what they believed to be feasible alternatives (Fig in S1 Answers). However, it may be debated what is feasible. In the Netherlands considerable difficulties hampered the implementation of the short metal chain between 1994 and 2003 [42, 43], and again to implement the EC Directive roughly between 2003 and 2011 [6, 44–47]. Given these experiences, and given the fact that non-EU countries generally do not provide any enrichment indicating it is mostly not economically feasible, it is unlikely that destructible or edible natural materials will be feasible in the near future.

**Fig 2:**
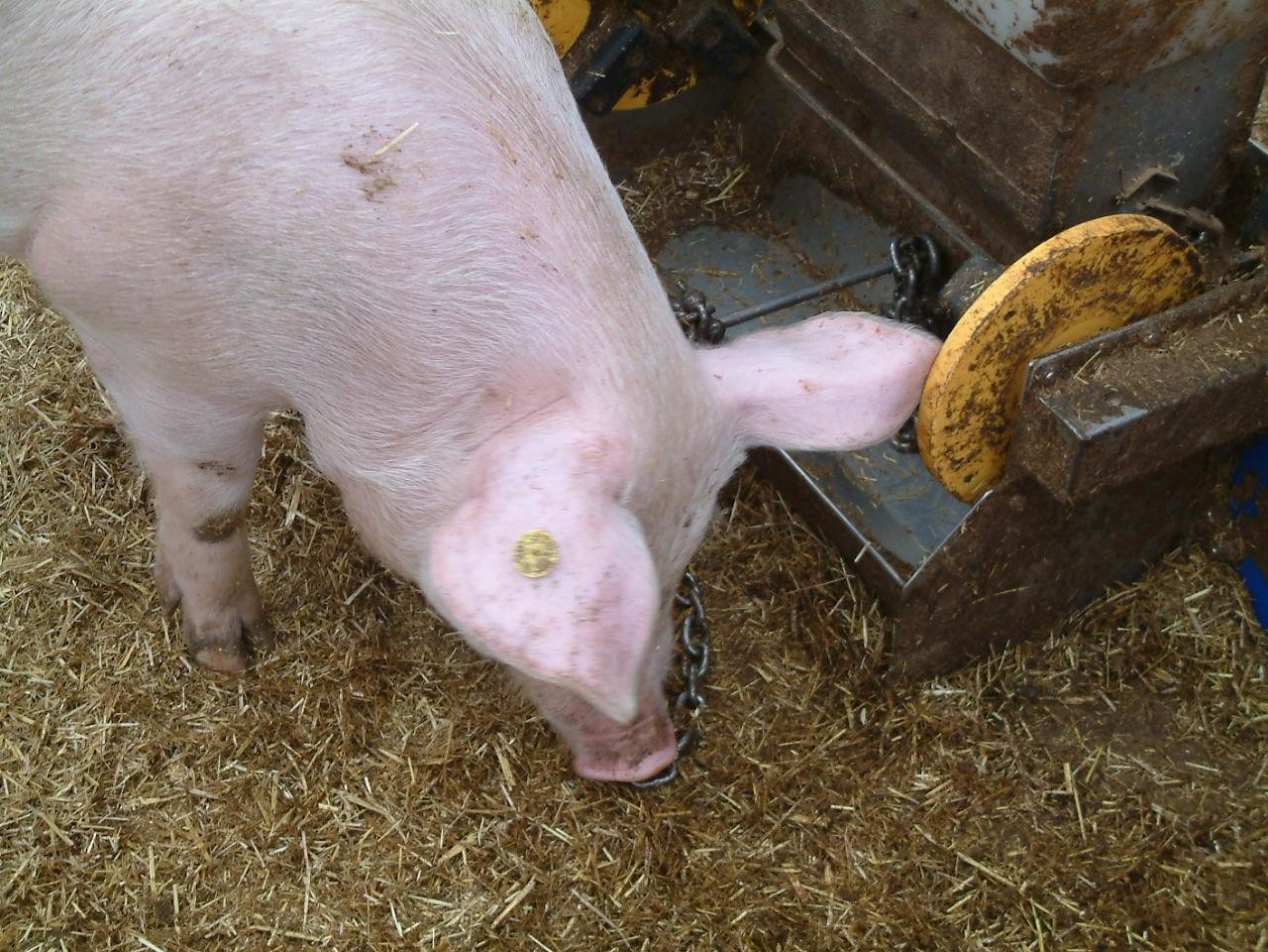
Pig manipulating a chain on the floor covered with straw. The feeder (actually a rooting bin) in the picture was permanently empty and not used for feeding or rewarding the pigs. Note that the chain is a stainless steel anchor-chain, which is an apparently preferred type of chain [1] (Photo by Herman Vermeer, reprinted with permission).

**Fig 3:**
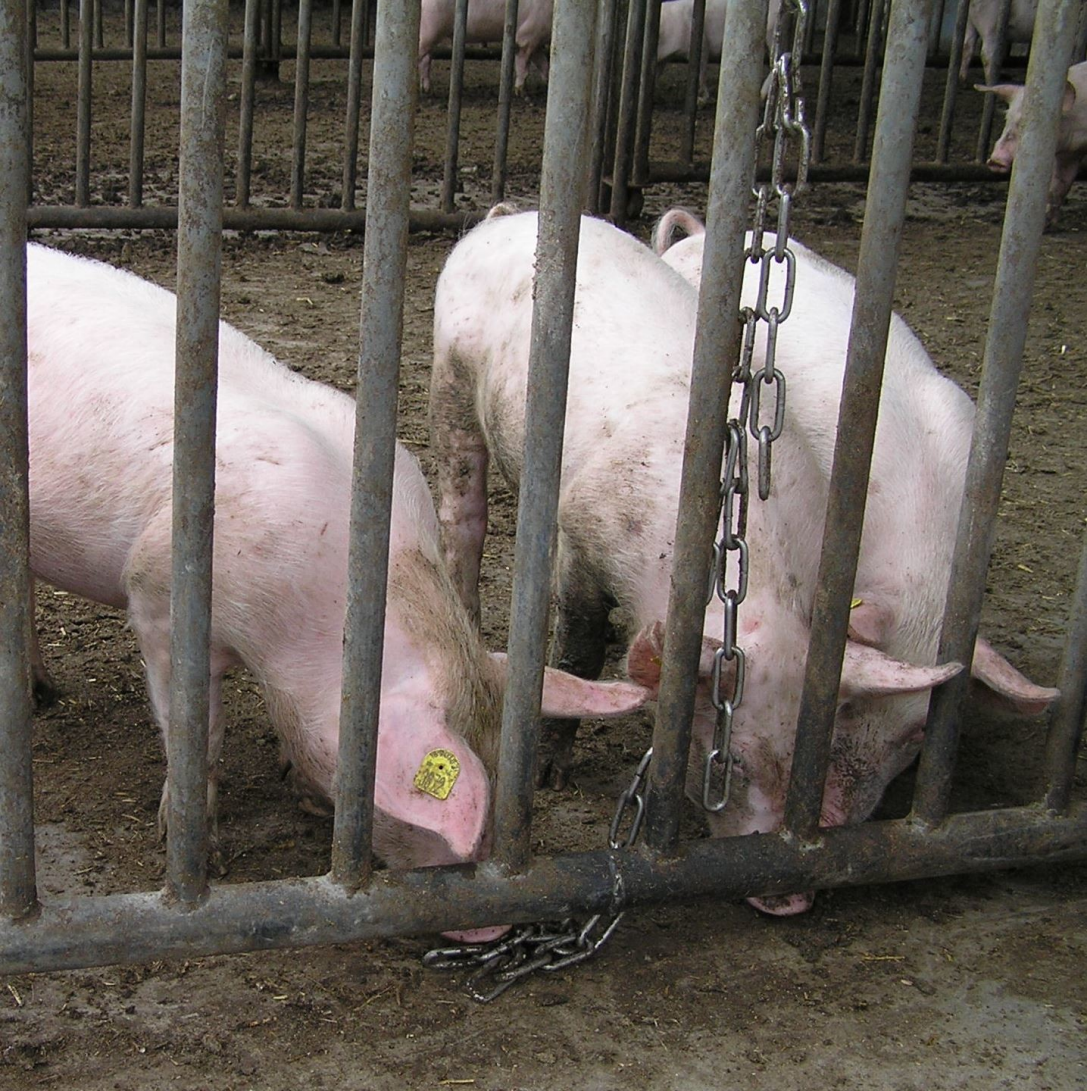
Branched chain. This is a chain reaching to floor level where the chain may be ‘rooted’ or manipulated while lying down, and to which two short pieces of chain have been attached such that ends of a chain are available at nose height to pigs of different sizes or age groups for manipulation while standing. Note, however, that this is a presumably inferior-type of c-chain, not a recommended stainless-steel anchor-chain.

**Fig 4:**
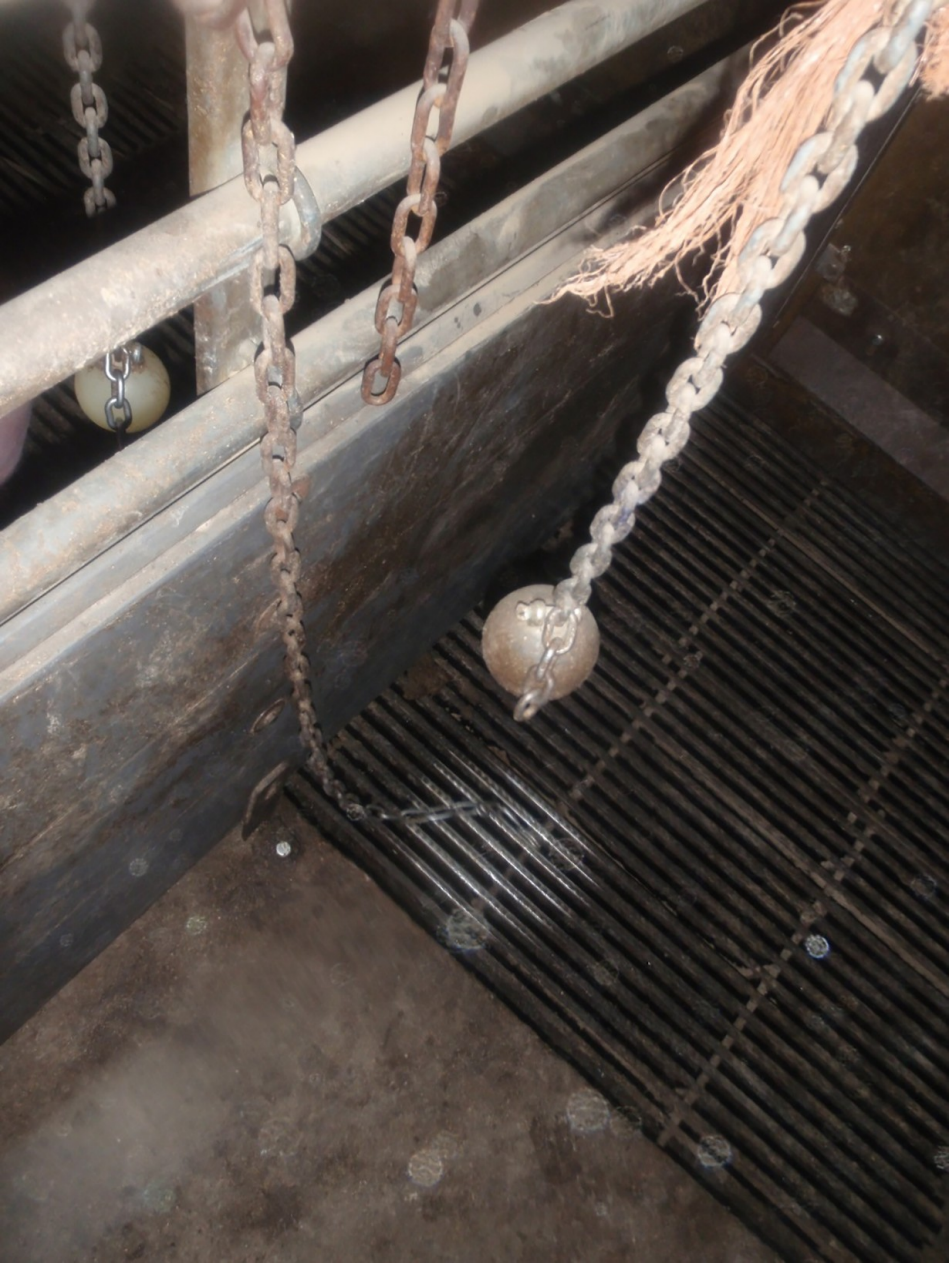
Balls dry and collecting dust near a short chain and a chain reaching to floor level. The short chain is hanging too high and rusty, and the metal slats are shining, indicating intensive use of the part of the chain that is resting on the floor.

Experiences in welfare schemes, such as the Dutch ‘Better Life’ scheme, also indicate a tendency to compromise welfare improvements for economic reasons. PVC pipes (called ‘strokokers’), for example, filled with pressed straw briquettes were introduced a number of years ago, but their contribution to pig welfare remains questionable [1]. Contrary to expectation, however, destructible materials do not always benefit pig welfare. When a hay rack was provided to pregnant sows, Vermeer (pers. comm.) observed more aggression and skin lesions probably because the rack was not sufficiently accessible. A similar observation (MB, pers. obs.) concerned the mixing of grass silage through the feed of organic growing pigs [48], while, conversely, Branched chains appear to be most valued by pigs even when they have access to rootable straw bedding (Figs 2–4; [1]). This means that Branched chains could be valuable, not only to improve pig welfare in a most feasible way, but also as a benchmark to validate whether proposed alternatives may qualify as ‘proper enrichment’. For this, the alternative enrichment should at least be shown to attract significantly more object manipulation than the Branched-chains design.

Taken together, without feasible alternatives Branched chains should be implemented widely in commercial pig production to avoid unnecessarily compromising pig welfare by perpetuating animal boredom, frustrating natural behaviours (like rooting) and behavioural needs (like exploration and foraging). The Branched-chains design may not itself be sufficient to raise pigs with intact curly tails, but it is the biggest first step that can be taken in the short term towards proper enrichment and the ultimate objective of eliminating abnormal behaviours like tail, ear and flank biting, reducing the need for tail docking to prevent tail biting, and thereby increasing the level of public acceptance of intensive pig production.

## Conclusions

This expert survey showed a (partial) rejection of the hypothesis that by adding indestructible materials to the short metal chain, the welfare of intensively farmed pigs had reduced across Europe. While experts generally gave slightly higher welfare scores for added indestructible synthetic pipe and (hard) wood, and slightly lower scores for small balls attached to the chain, the overall welfare benefits of adding indestructible objects were negligible and well below the level the experts, mostly welfare scientists, considered acceptable. Improving the short metal chain by making it longer, reaching to floor level and providing more chain ends (e.g. 1 branched chain with 2–3 chain ends at variable heights per 5 pigs) would significantly and considerably improve pig welfare, almost (but not quite) sufficiently to reach the experts’ cut-off point for acceptability. As such it seems to be the ideal starting point for further improvement, e.g. by adding an indestructible object, and a most feasible benchmark for improving pig enrichment world-wide [1].

## Supporting information

S1 Dataset Anonymised expert responses. Pink cells have been deleted to maintain expert anonymity.
(XLS)

S1 Survey The invitation to participate and the questionnaire (table) with introductory text. (DOCX)

S1 Answers Summary of responses to questions Q3 and Q11-25. (DOCX)

S1 Table Prevalence of metal chains and hanging indestructible objects in various countries/regions according to expert respondents (one row per respondent). (DOCX)

S2 Table Final REML model for the transformed welfare scores of enrichment materials. (DOCX)

S1 Fig Enrichment in 841 pens on 47 Dutch conventional pig farms in 2011. (DOCX)
