# Supplementary material for: Expert opinion on metal chains and other indestructible objects as proper enrichment for intensively-farmed pigs
Source: PLoS One. 2019 Feb 22;14(2):e0212610. doi: 10.1371/journal.pone.0212610 (PMC6386313; doi:10.1371/journal.pone.0212610)
Supplement: S1 Survey — (DOCX) [file pone.0212610.s002.docx]

**S1 Survey.**

**Table. The invitation to participate and the questionnaire (table) with introductory text.**

**Invitation to participate:**

Dear all,

Presently, I’m reviewing enrichment materials for intensively-farmed pigs and I've made a brief questionnaire for scientists, veterinarians and farm advisors with an academic background and extensive practical experience.

My focus is on the provisioning of chains and indestructible objects for esp. weaners and growing pigs kept in conventional pig farms. Materials such as pipes, balls and hardwood have been attached to metal chains on pig farms in Europe to meet the EC Directive 2001/93. This directive states that all pigs must have permanent access to a sufficient quantity of material to enable proper investigation and manipulation activities. I'm interested in publications, personal observations and (empirically-based) opinions on the value of these materials for pigs. All responses will be treated confidentially.

Would you be as kind as to have a look at my questionnaire?

Many thanks for your consideration,

Marc

**The questionnaire:**

Presently, I’m reviewing enrichment materials for intensively-farmed pigs. Below you find a brief questionnaire for experts, i.e. scientists, veterinarians, farm advisors with an academic background and extensive practical experience.

I need help because this is an important as well as problematic issue. My focus is on chains and indestructible objects for esp. weaners and growing/fattening pigs kept in conventional pig farms (i.e. excluding pigs in specific welfare schemes or organic farming). Indestructible objects hanging on a chain are widely used in Europe esp. following the implementation of EC Directive 2001/93 stating that all pigs must have permanent access to a sufficient quantity of material to enable proper investigation and manipulation activities. My own research involving reviewing, modelling and on-farm work on this subject (see eg "Bracke enrichment pigs" in Google Scholar) may have been at the basis of the political decision in the EU that chains alone were no longer considered to be sufficient. This led many farmers in (mainly N-W) Europe to attach to the chain a piece of rather indestructible plastic pipe, ball or (hard)wood. Unfortunately, I'm also (next to) convinced that as a general rule this implementation may in fact have reduced pig welfare. Therefore, I need your help to either confirm or refute my hypothesis, as well as to specify what would be adequate enrichment.

Below you find a list of questions in a table. Please provide answers only to questions you feel sufficiently knowledgeable about. All answers will be treated strictly confidentially.

For further processing please insert your answers as much as possible in the cells of the table as specified. Grey cells should not be used.

Many thanks for your answers and your time. It’s highly appreciated.

Most kind regards,

Marc Bracke, 15-2-2016.

| R | **Question** | **Column A** | **B** | **C** | **D** |
| --- | --- | --- | --- | --- | --- |
| 1 | Which country(s)/geographical area are you knowledgeable about? Please specify using 2 character-codes, if possible, e.g. NL, UK, FR, US, CA). |  |  |  |  |
| 2 | What is your primary response to my hypothesis that as a general rule the welfare of intensively-farmed pigs has been reduced by adding an indestructible object to (the end of) the metal chain? Please specify in column A the extent to which you agree (0 totally disagree - 10 totally agree; NA if unknown); Specify in column B the extent to which you believe this is the general perception in practice, i.e. among farmers, veterinarians and extension (score 0 totally disagree - 10 totally agree). You can use columns C and D to add a brief comment (e.g. why) to the answers provided in columns A and B respectively. | Score: | Score: | Namely/Because: | Namely/because: |
| 3 | Do you have personal observations or referenced sources of information either supporting or refuting my hypothesis? Please provide answers in columns A and B respectively, and a short explanation (optional) in Column C. You may use an annex for a more lengthy answer at the end of the table (Row 24). | Support: Y/N | Reject: Y/N | Namely: |  |
| 4 | What is the prevalence of metal chains (without further objects attached) in conventional pig pens in your country/area? Note: A rough estimate suffices. Use column A for your main answer. Specify NA (not available) in Column A if you don't know. Optionally: specify a range (from - to) in the columns B and C respectively. You may specify values for different countries as follows, e.g. UK: ...; DE: ....; ES: ...... |  | From: | To: |  |
| 5 | What is the prevalence of metal chains supplemented with one or more indestructible object attached to it? |  | From: | To: |  |
| 6 | What is/are the main indestructible objects attached to chains in your country? Give up to 3 suggestions listed in order of presumed prevalence in columns A to C. |  |  |  |  |
| 7 | How prevalent are these mainly used objects? The answers in columns A-C correspond to what was specified in the previous question. |  |  |  |  |
| 8 | In your opinion, how good are the main (chains with) indestructible objects for pig welfare on a scale from 0 to 10, i.e. compared to a pen without any enrichment and compared the best possible enrichment respectively. Please use a score of 5.5 as the cut-off point for what you consider to be adequate/sufficient enrichment for pigs. Please also specify your welfare score for the chain without any attached objects in column D. The answers in columns A-C correspond to what was specified in rows 6 and 7. |  |  |  | Chain: |
| 9 | What welfare scores would you give to the following indestructible objects attached to the end of the chain at nose height of the pigs: A: Pipe; B: Hockey-size ball; C: Big ball (size: small football); D: (Hard)wood (Note: all objects are more or less indestructible/very long lasting). | Pipe: | Small ball: | Big ball: | Wood: |
| 10 | What scores would you give to the following types of chain: A. 1 chain for up to 15 pigs with the chain ending at nose height of the smallest pigs entering the unit; B: as A but hanging too high for a large majority of the smallest pigs; C: as A but reaching to floor level (solid floor) such that pigs can also 'root' the end of the chain and can manipulate the chain while lying; D: as A but now 1 chain for every 5 pigs (such that more pigs can play at the same time), and chains ending at various heights, including both nose height and floor level. | 1 chain/15 pigs: | Too high: | On floor: | 1 chain/5 pigs & at various heights: |
| 11 | Do you have specific recommendations for further optimising the use of metal chains with/without indestructible objects attached to it? Please specify up to 3 aspects in columns A-C respectively, and indicate in column D what the (improved) welfare score would be if all your recommendations were implemented. Leave blank if you are pressed for time. Use NA if unknown. |  |  |  | Score: |
| 12 | Conversely, do you have specific recommendations to avoid welfare reduction/inadequacies when providing chains with/without indestructible objects? Please specify up to 3 aspects in columns A-C, and indicate in column D what the (reduced) welfare score would be if all your recommendations were implemented. Leave blank if you are pressed for time. Use NA if unknown. |  |  |  | Score: |
| 13 | Which feasible enrichment (object/substrate) would you recommend in order to further improve the welfare of conventionally-housed pigs? If any. Specify up to 4 suggestions in columns A-D. Use NA in column A if unknown. |  |  |  |  |
| 14 | What welfare score(s) would you give to the suggested improvements respectively? |  |  |  |  |
| 15 | What are your main considerations for the scores given? Leave blank if pressed for time. E.g. state a few keywords in columns A-D. You may provide more text in Row 25 as Annex to this question. You may use a formal notation using the following abbreviations. Please consider aspects like animal-material interactions (AMI, e.g. biting (b), chewing (ch), rooting (r)), pen-directed/penmate-directed behaviour (pdb, pmdb), tail/ear/flank biting (tb, eb, fb), and other aspects such as material degradation (md), health (h), pen soiling (ps), frustration (f), aggression (a), abnormal behaviour (ab) and stress (s), but also cognitive bias (cb), natural behaviour (nb) and preference strength/demand (ps/d). Information may be based on e.g. personal observations (po), report (rep) or a scientific reference (sciref). Explain main differences between scores by referring to objects using question numbers and column labels (e.g. 9A = pipe). For example: 8D<9A:AMI,rep. means: chains (8D) are used (AMI) less than (<) pipes (9A) according to a report (rep). For specific/less well-known reports and scientific references it is highly appreciated if you could provide reference details (in Row 25). But again, no worries if you skip this question. |  |  |  |  |
| 16 | How knowledgeable are you about “pig welfare and enrichment” (give a score 0-10, compared to all other scientifically-trained people in the world who think they know something about this subject). Please state your type of expertise in Column B. | Score: | Scientist/Vet/Advisor/Other |  |  |
| 17 | Who are the most knowledgeable experts in the world on this subject in your opinion? You may specify up to 4 experts, preferably ranked in the order of expected expertise in columns A-D. |  |  |  |  |
| 18 | Please provide contacts (names & emails/phone) of other experts who should be contacted because they have specific expertise. This concerns esp. experts with relevant practical experience e.g. veterinarians and farm advisors with particular interest in this subject. |  |  |  |  |
| 19 | May I contact you again in the future, e.g. for further questions/explanations? | Y/N |  |  |  |
| 20 | May your name be listed in the acknowledgements? | Y/N |  |  |  |
| 21 | Would you be interested in becoming a co-author of this paper? (But please note that showing interest does not automatically imply co-authorship). | Y/N/Perhaps |  |  |  |
| 22 | Do you wish to be kept updated (e.g. about the results and publication)? | Y/N |  |  |  |
| 23 | Do you have any other information that you consider relevant? This concerns information that is unlikely to be retrieved using a regular literature search, e.g. because it is published in another language. In particular, I’m looking for personal observations and unpublished reports, or other publications and important arguments/considerations (on any type of pig) that may otherwise get overlooked. If you want you are welcome to provide short descriptions of your own main findings that I can copy/paste into my more general review about enrichment for intensively-farmed pigs. (Providing concise descriptions may actually increase the probability of getting quoted, :-).) |  | | | |
| 24 | Annex to Question 3 (information about confirming/rejecting the hypothesis) |  | | | |
| 25 | Annex to Question 15 (explaining welfare scores given) |  | | | |
| 26 | Any other comments/suggestions? |  | | | |

Many thanks. Please send to: [marc.bracke@wur.nl](mailto:marc.bracke@wur.nl).
