# Supplementary material for: Expert opinion on metal chains and other indestructible objects as proper enrichment for intensively-farmed pigs
Source: PLoS One. 2019 Feb 22;14(2):e0212610. doi: 10.1371/journal.pone.0212610 (PMC6386313; doi:10.1371/journal.pone.0212610)
Supplement: S1 Answers — (DOCX) [file pone.0212610.s003.docx]

**S3 Answers.**

**Summary of responses to questions Q3 and Q11-25.**

**Q3. Do you have personal observations or referenced sources of information either supporting (3a) or refuting (3b) my hypothesis?**

3a. 12 affirmative and 11 negative answers were received. 3b: 5 affirmative and 13 negative answers. In total 21 publications were suggested, and 23 clarifications. The latter were mostly personal observations (see the List below).

Several publications were referred to. These included Broom and Fraser [11] (pp. 252; 258; 263; 297-307); Fraser [12], Fraser et al. [13], Feddes and Fraser [14], Telkänranta et al. [15], Hothersall et al. [16], Van de Perre [17], Bulens et al.[18], Permentier [19], Courboulay [20, 21], [21], Courboulay and Thuard [22], Grandin [23], Pol et al. [24], EFSA [25, 26], EC [27-29] and Caille and Loussouarn[30], Larsen et al. [31], Nannoni et al. [32] and Zonderland and Zonderland [33].

**List. Expert responses to the question whether they had personal observations or referenced sources of information either supporting or refuting the hypothesis that as a general rule the welfare of intensively-farmed pigs has been reduced by adding an indestructible object to (the end of) the metal chain.** Different alphabetical characters refer to different experts. In square brackets, a brief author response note has been provided.

**Referenced information supporting the hypothesis**:

A: Even when a polythene pipe cross on a chain did elicit more object use than a plain chain, it did not reduce tail biting [15]. [Note MB: such a pipe cross is not common on commercial farms in the Netherlands, where much smaller pieces of pipe are used and where destroyed pieces of pipe do not always get replaced. Furthermore, in the study the object location, object size and number of objects per treatment were confounded.]

E: We tested objects attached to a chain (for example balls), pigs tended to play with the chain and not with the object (the objects sometimes served as a "stopping point" when pigs are dragging the mouth along the chain) [21].

**Anecdotal observations supporting the hypothesis**:

A: When suckling piglets are provided with either a simple chain or a commercial hard plastic ball suspended on a chain, they manipulate the ball even less than the simple chain.

B: Personal observation in commercial farms

C: Personal observations of farms where such objects were not being used by pigs.

I: Vets/farmers tend to think that indestructible object and/or chains do not work against tail biting. Pieces of hard wood added to a metal chain do not stimulate much manipulation. Can bother the pigs depending on the situation of the material in the pen. However, some farmers using plastic pipes and other objects hanging from "moving chains" are happy with this.

L: I feel most people believe if you just stick something up there then it improves enrichment. Most enrichment is chosen to please the human, not the animal. I have seen pigs play with chains but not the other items. I think a pipe they could pick up would be better than attached to a chain. The wood may have some benefit if they can chew it and "destroy" to some level. For my most recent study we used the PorciChew which would be like the chain but better, and we had a large ball for them to root and roll around. They didn’t play with these items as much as I had hoped. But they did use them. Sometimes they would walk by the chain and if it hit them they’d stop to play with it. They’d stand on the ball more than root it around it seemed. When I first started working with pigs we collected old bowling balls from bowling alleys, to combat tail biting in finishers. They played with them initially, but the balls quickly got dirty and shoved between the fence and feeder where they could no longer use them. If they could not get stuck in the pen and feces were cleaned off daily, I think they would have used them longer. Taking them out and reintroducing them also keeps interest. In the nursery pig study, what we found they loved the most was something they could destroy. We put in old feed/bedding bags and that was probably their favorite item because they could tear it apart. We also did a pan with peat moss, soil, and shredded paper. Neither our farm or lab would ideally allow for straw so we used other forms of substrate that could be washed down the drain without causing any issues. As soon as that enrichment was changed it was the first thing they would go to and play in and root out of the pan. They went from clean little pigs to completely covered in dirt! A few other non-destructible things we used were dog toys (kongs, footballs, ropes, DNA flex, rubber boot) and they would pick these up and chew and toss them or get treats out of them. We put in a towel which they would lie on and carry around in their mouth. They mouthed it and drug it around, but never destroyed the towel to my surprise. They had access to enrichment continuously but from personal observations it seemed like they interacted the most with it when it was changed.

**Referenced observation opposing the hypothesis:**

H: [2]. [MB: This study does not refute my hypothesis.]

**Anecdotal observations opposing the hypothesis**:

A. There are some indestructible objects that do seem to increase object use (though increased object use does not always automatically mean increased welfare) when added to the end of a chain. In Finland, some farmers have started suspending ice hockey pucks (hard rubber) on a chain, and especially weaned piglets seem to use them frequently over several weeks. Pieces of polythene pipe in a chain also seem to elicit more manipulation than a plain chain, if the pipes are attached in a horizontal position. There is also the question as to what counts as indestructible: dry wood is almost indestructible but also quite uninteresting, while recently harvested wood is either indestructible or slowly destructible depending on the tree species and the age of the pigs it is given to. I anecdotally interviewed a farmer. He said that the pigs' interest in fresh birch wood pieces remained high for the two first batches of finishing pigs, i.e. approx. 7 months after attaching them to the pens (9 months after felling the trees); after this, the interest was rapidly lost, but the pieces were still not destructed. When fresh these pieces of birch reduced tail biting significantly. Birch wood is a hardwood species with a sweet taste due to its high xylitol content. Since the birch wood was not completely destroyed after the first 2 batches, it can be classified as indestructible and may thus be a rare case of indestructible objects that do increase welfare when added to chains.

D: I have observed use of minimally destructible material that has a minor positive effect on pig welfare. I do not think chains are very effective at any height or location. Of course pigs like to root, so one might think that on the floor they will root more than when hanging. But on the floor, the chains become dirty and they lose interest. So although the floor accommodates the natural rooting behavior, the hanging chains remain cleaner and may be used less at first, but more over time. Not all pigs use the chains, but a few become obsessed with them. In the USA, people hang chains when they have tail biting. But they do not stop the tail biting. However, adding straw or soil for rooting will reduce the tail biting. But then people have issues (here) with the manure pits – they can’t handle rooting materials easily. If 1 pig out of 10 enjoys manipulating a chain, should this be provided? Or should we find an enrichment substrate that accommodates the interest of 9/10 pigs? The latter of course would be preferred. In the USA, apart from specialty markets, large farms are driven almost entirely by economics. Adding enrichment is a cost with no financial return. So like Facebook says for relationships; its complicated.

F: Personal observations (not specified); I don't think it has reduced welfare, just not improved.

G: I have recently seen pigs in China using logs attached to a chain well (but logs loose on the floor are used much less).

J: I think adding an extra object to the chain is slightly better than nothing, as the pigs can manipulate the object & chain [Note from the author, MB: I used to think so too, but I now know this is false in the prevalent case that the object is attached to the end of a short chain and hung rather high to reduce destructibility of the object.]

J: We have tried some 'home made' enrichment, pigs seemed to use the attached object more often than only the chain. In some cases a chain is not used at all; a ball, chain or pipe on two sides of a pen was used more; a chain in the dunging area is not a good idea.

K: I don't have much experience of indestructible objects on chains except on our own unit, where plastic (alkathene) piping is very well chewed. I don't know if you call this "indestructible". [MB: I expect that well-chewed pipes are destructible and hence will need regular replacement, which is often less of a problem on an experimental farm compared to commercial farms.]

Note MB to all ‘refutations’: All suggestions are compatible with the proposed hypothesis.

**Q11. Do you have specific recommendations for further optimising the use of metal chains with/without indestructible objects attached to it? Also indicate what the (improved) welfare score would be if all your recommendations were implemented.**

Suggestions were variable and collectively included the following suggestions for improvement: Destructibility (e.g. rope, soft wood, straw), (but also!) indestructibility (robust, not broken), edibility (plant-based, straw), rootability (e.g. chains holding some basket to root treats out of), chewability (e.g. softer rubber hose; PorciChew (Ketchums, UK)), smell, taste, mouth feel (e.g. size of the links of a chain), shape, size (e.g. various link sizes), complexity, object movability (e.g. also by pigs in neighbouring pens), variability (e.g. change every other day), additions (e.g. screws, side chains), accessibility (for multiple pigs to explore; keep the end of the chain accessible when adding objects; longer chains; nose height & till floor level; number of chains/pig), hygiene (not in the dunging area; cleaning, disinfection), location in the pen.

The average welfare score for complying with the recommendations as suggested by the expert was 7.1 (S.E.: 0.6; n=8).

**Q12. Conversely, do you have specific recommendations to avoid welfare reduction/inadequacies when providing chains with/without indestructible objects? Please also indicate what the (reduced) welfare score would be if all your recommendations were implemented**.

Suggestions to avoid inadequacies referred to: location (not in a corner; too close to drinkers, over the resting area), soiling (not in the dunging area; not loose on/close to the floor), attachment, indestructibility (hard materials), not enough (e.g. only 1 object per pen), objects too big to allow oral manipulation, risk for injury (e.g. by swinging objects), not chewable, monopolisable by a single pig, not in the resting or eating area, improper succession of objects (decreased attractiveness from weaners to fattening pigs), too few objects/pigs (competition), inadequate accessibility (e.g. too high), no novelty (incl. no rotation), blockage of the manure handling system.

As to the latter point, one US expert stated: “Paper, rubber and wood will not work in the US with the types of manure handling systems we have. Anything that falls into a pit needs to be dealt with!”. ... With the level of caretakers we have over in the US working in the barns, I am not confident that they would care or watch the wood being whittled down and then remove before it falls through the floor. The other huge concern with wood (or any material) is how we can disinfect (we call it fogging) the material before bringing it onto the farm to meet the biosecurity needs. With PRRS and PEDv and now Seneca virus, our biosecurity rules have continued to increase.”

Also: “The type of balls used here on farm have been heavy bowling balls. They weigh at a minimum 8 lbs. (3.6 kg). The pigs will roll them to the wall, roll then up the wall to about head height and release. This results in a heavy ball crashing back down onto concrete floors and causing holes or cracks in the floor. This is certainly not sustainable for producers to use!”

Another expert pointed out that metal chains reaching to floor level may get stuck, but also that this problem may be solved by using larger chain links or some larger items (screws, pieces of wood, rope, etc.) to the end of the chain.

When specified inadequacies were present the average welfare score was 4.3 (S.E.: 1.4; n=5).

**Q13, Q14 and Q15. Which feasible enrichment (object/substrate) would you recommend in order to further improve the welfare of conventionally-housed pigs? (Q13). What welfare score(s) would you give to the suggested improvements respectively? (Q14). And, what are your main considerations for the scores given? (Q15).**

The answers have been summarized in the Table and Fig below. The answers to Q15 largely overlapped with the relevant properties formulated in response to Q11 and Q12.

**Table. Clusters of enrichment materials considered feasible in conventional pig farming (including average welfare scores, standard deviation and number of respondents (N)).**

| **Enrichment considered feasible by the experts** | **Average welfare score** | **S.E.** | **N** |
| --- | --- | --- | --- |
| Straw; full bed of straw; straw bed on the floor | 8.38 | 0.55 | 4 |
| Bedding on solid areas (in combination with slats) | 9.00 |  | 1 |
| Straw rack(s); rack with long straw | 8.75 | 0.95 | 4 |
| Straw dispenser/straw pipe; handful of straw/hay/maize silage; straw/hay/silage in racks; ad lib compressed or pelleted straw in feeders; substrate dispenser; hay rack with narrow gaps | 7.92 | 0.27 | 6 |
| Compound materials: Combination of jute sack, (handful of) straw/hay/maize silage, and sizal rope, and variably provided; Straw, sand or other maleable materials; Straw, wooden twigs, other fibrous material; Ropes and straw/alfalfa; Combination of fresh, horizontal birch wood, jute sack and roughage in a basket, preferably also with two times a day roughage given on the floor; Straw, jute, rope; Straw, peat, (straw + silage (or other roughage)) | 7.79 | 0.50 | 7 |
| Peat/turf | 7.25 | 0.75 | 2 |
| Other substrate (than straw); Organic fibrous material on the floor; Substrates: They might have potential after chopping and drying various types of plant waste from agriculture (stems, leaves etc.) and experimenting whether these could be given in slatted-floor pens in larger quantities as compared to straw, as the use of straw is usually limited by the risk of obstructing the slurry system; Basket with roughage; Something organic that the farmer wants to renew every day; Any edible, rootable substrate; Substrate to root (shredded paper dissolves); Ingestible compacted materials (e.g. by products containing high levels of dietary fibre) | 7.94 | 0.50 | 8 |
| Working for food: Devices dispensing dry feed, with designs with which pigs have to work in order to attain the feed; Work for feed: balls or chains in trough; Destructible wood with piece of feed inside | 7.50 | 0.50 | 2 |
| Soft wood: Pieces of trees harvested within the last couple of months and used for max half a year at a time, so they still retain the smell, taste and mouth feel resembling living plants. Differences between tree species to be taken into consideration include eg hardness, xylitol content (sweet taste), volatile organic compounds (in conifers, can be mildly harmful) etc. The attractiveness largely also depends on object design, such as providing pieces horizontally and making them thin enough so the pig can take it into the mouth and chew with molars of both sides (diameter of 5-10cm for finishing pigs and 3-5cm for weaned piglets); Soft wood branches of thin diameter; Fresh birch wood attached horizontally; Branches- edible and destructible can be used in slatted floors (score is when replaced frequently); Fresh wood hanging from e.g. chains | 5.60 | 0.40 | 5 |
| Rope(s); sisal rope; organic hanging rope; hemp rope; destructible rope | 6.60 | 0.68 | 5 |
| Jute (or sisal rope); Jute sack; Jute sack (high quality, attached to pen wall, tightly hung (not like a rope) | 6.67 | 0.88 | 3 |
| Objects on the floor: Small wood blocks fixed to the floor or at least touching the floor; Large wooden log fixed to the floor; Any enrichment on the floor (the ball and wood would work here too); Loose manipulable objects; Plastic pipes (deformable) fixed to the floor or at least touching the floor; Chains fixed to the floor or at least touching the floor | 7.70 | 0.36 | 6 |
| Rubber boots when they get too worn out to wear | 8.00 |  | 1 |
| Floor feeding with abundant space available | 7.00 |  | 1 |
| Newly engeneered material specifically designed for indestructibility or slow destructibility, using biochemical and chemical-engineering know-how of new biorefineries, and using waste products of the timber and paper industry as raw materials; tailoring the smell, taste, mouth feel, hardness etc. according to the pigs' preferences as well as the practical demands of the farmers; Manipulable hanging object with properties: chewable, e.g. soft wood; destructible e.g. rope; edible, e.g. plant material or straw | 8.50 | 0.50 | 2 |

**
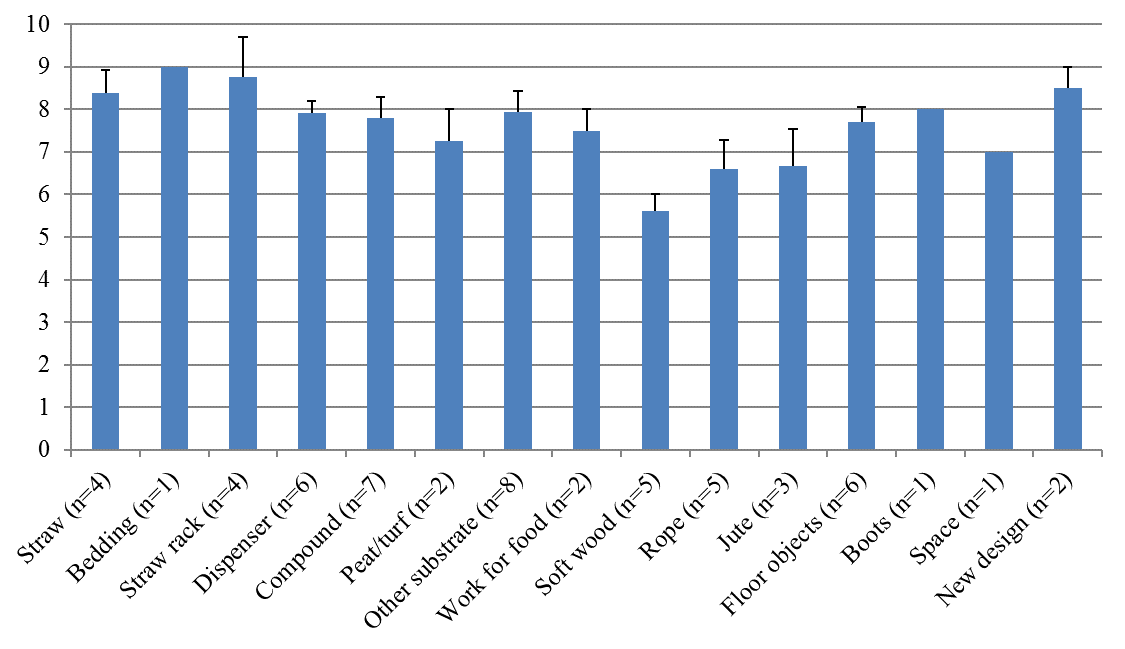
**

**Fig. Average welfare scores (and standard errors) of clusters of proposed feasible enrichment materials for conventional farming (see more detailed descriptions in the Table above).** N: number of respondents.

**Q16. How knowledgeable are you about “pig welfare and enrichment”, and what is your type of expertise?**

The average self-reported knowledgeability score was 7.6 (S.E.: 0.3; n=27). In total 29 respondents specified one or more types of expertise: 28 were scientists, 5 had veterinary expertise (as well), 4 had advisory qualifications and 4 classified as ‘other’ (e.g. as animal keeper).

**Q17. Who are the most knowledgeable experts in the world on this subject in your opinion?**

Sandra Edwards was considered the most knowledgeable (n=11), followed at a distance by myself (n=4), Bo Algers (n=3), Heleen van de Weerd (n=3), Valery Courboulay (n=3), Jonathan Guy (n=2), Beat Wechsler (n=2) and Nina Taylor (n=2). Another 14 experts were mentioned once. Farmers were mentioned 4 times as a group. Vets 4 times, but once it was stated that their state of knowledge is overrated. This question was included to verify whether the main experts had been contacted. Of the identified most-knowledgeable experts 11 also responded to the questionnaire, 2 were contacted without success, and 8 were not contacted.

**Q18. Please provide contact details of other experts who should be contacted.**

In total an additional 21 experts were invited to participate. This question enhanced the probability of adequately identifying experts as experts.

**Q19. May I contact you again in the future?**

Yes: 25; No: 1.

**Q20. May your name be listed in the acknowledgements?**

Yes: 23; No: 2.

**Q21. Would you be interested in becoming a co-author of this paper?**

Yes: 9; Perhaps: 11; No: 6.

**Q22. Do you wish to be kept updated?**

Yes: 26; No: 0.

**Q23 and 26. Do you have any other information/comments that you consider relevant?**

The further information provided by the experts was incorporated in the answers presented above and in the discussion.

**Q24 and 25**: This were not separate questions, but merely cells providing extra writing space in the questionnaire to answer Q3 and Q15.
