# Supplementary material for: Expert opinion on metal chains and other indestructible objects as proper enrichment for intensively-farmed pigs
Source: PLoS One. 2019 Feb 22;14(2):e0212610. doi: 10.1371/journal.pone.0212610 (PMC6386313; doi:10.1371/journal.pone.0212610)
Supplement: S1 Table — (DOCX) [file pone.0212610.s004.docx]

**S4 Table.**

**Table. Prevalence of metal chains and hanging indestructible objects in various countries/regions according to expert respondents (one row per respondent).**

| **Country** | **Prevalence of (bare) metal chains** | **From** | **To** | **Prevalence of indestructible objects (on chain)** | **From** | **To** | **Most prevalent indestructible object** | **Prevalence** | **Next most prevalent indestructible object** | **Prevalence** | **Third most prevalent indestructible object** | **Prevalence** |
| --- | --- | --- | --- | --- | --- | --- | --- | --- | --- | --- | --- | --- |
| UK |  | 0 | 5 | Note: 50% of all pens in the UK have no straw. All of these have a chain with hardwood. The other 50% of pens have straw. 20% of these pens also have a chain with hardwood (the % with chains in these pens may be an overestimate - it's not high) | 50 | 70 | Hardwood on chain-specified by farm assurance | 60 |  |  |  |  |
| UK | NA, but since not allowed in UK Red Tractor farm assurance (>95% of farms) likely to be low | 0 | 5 | ~50%, i.e. the majority of the pigs not in straw based systems | 30 | 60 | Plastic pipe |  | Wood |  |  |  |
| UK & others | Common |  |  | Common |  |  | Metal bar |  | Plastic bar |  |  |  |
| UK & DK |  |  |  |  |  |  | Hard wood | 70 | Plastic pipe | 20 |  |  |
| UK & DK |  | 20 | 40 |  | 60 | 80 | Hard wood that is too big for the pigs |  | Balls |  |  |  |
| NL | 80 |  |  | 90 |  |  | Polyethylene pipe | 70 | Hose | 10 | Wood | 10 |
| NL | 20 | 5 | 35 | 70 | 50 | 90 | Ball | 50 | Piece of wood | 20 | Plastic pipe | 15 |
| NL |  |  |  |  |  |  | Balls |  | Plastic rings around bars for sows | High in farrowing pens |  |  |
| NL |  |  |  |  |  |  | Plastic ball |  | piece of wood |  | Piece of plastic (e.g. pipe) |  |
| NL & BE |  |  |  |  |  |  | Piece of wood |  | Plastic ball |  | Pipe |  |
| BE | 90 |  |  | < 5% |  | 5 | Plastic/rubber bite tubes/balls | 1.6 | Ball with a smell | 1.6 | Bite-rite | 1.6 |
| [DE] | 10 |  |  | 20 |  |  | 6cm superhard plastic ball | 60 | Plastic form | 40 |  |  |
| FI | 80 |  |  | 20 |  |  | Piece of wooden board, dry & several yrs old | 16 | Hard plastic ball | 2 | Ice hockey puck | 2 |
| FI & EU | Very frequently used. |  |  |  |  |  | Dry wood |  | Plastic balls or other plastic objects |  | Ice-hockey pucks |  |
| SE | 0% (all pigs have access to straw) |  |  |  |  |  |  |  |  |  |  |  |
| SE | Low. All farmers use straw or straw or in rare cases only saw dust. Chains not commoly used, and if used, they are only used when there have been tail biting. Chains are then used as an extra toy for the pigs (esp. when tail biting) |  |  | Not seen |  |  |  |  |  |  |  |  |
| FR | High (Many farmers use chains without objects) |  |  | Low (few) |  |  | Plastic ball or toy |  |  |  |  |  |
| FR |  | 60 | 70 |  | 20 | 30 | Tough ball | 20 |  |  |  |  |
| IT | 30 |  |  | 5 |  |  | Wood |  |  |  |  |  |
| IT (NE) |  |  |  |  |  |  | Wood |  | Plastic |  |  |  |
| IT (North) |  |  |  |  |  |  | Wood |  | Plastic object |  | Salvaged material |  |
| ES |  | 20 | 30 |  | 1 | 3 | Pipes at head height | 1 | Hard wood at floor level | <1% | Plastic cans at head height | <1% |
| ES |  | 5 | 40 |  | 0 | 15 | Ball | 5 | Plastic tube | 10 |  |  |
| US | < 25%; mostly in summer when tail biting is higher |  | 25 | Near 0 |  | 5 | Rubber garden hose; but it is destructable over time | Rare; < 10% |  |  |  |  |
| US | 5 |  |  |  | 0 | 5 | [None used that I am aware of] | 0 |  |  |  |  |
