# Supplementary material for: Expert opinion on metal chains and other indestructible objects as proper enrichment for intensively-farmed pigs
Source: PLoS One. 2019 Feb 22;14(2):e0212610. doi: 10.1371/journal.pone.0212610 (PMC6386313; doi:10.1371/journal.pone.0212610)
Supplement: S2 Table — (DOCX) [file pone.0212610.s005.docx]

**S6 Table.**

**Table . Final REML model for the transformed welfare scores of enrichment materials.**

| **Source** | **Numerator df** | **Denominator df** | **F** | **Sig.** |
| --- | --- | --- | --- | --- |
| Intercept | 1 | 21.402 | 321.152 | 0.000 |
| Enrichment material | 8 | 15.181 | 33.157 | 0.000 |
| Gender | 1 | 21.402 | 6.364 | 0.020 |
| Region | 3 | 21.29 | 5.494 | 0.006 |
| Enrichment * Gender | 8 | 15.181 | 3.042 | 0.030 |
| Enrichment * Region | 24 | 15.562 | 3.785 | 0.004 |
| Enrichment * Gender * Region | 27 | 15.99 | 2.223 | 0.049 |

Sig.: Significance.
