# Supplementary material for: Expert opinion on metal chains and other indestructible objects as proper enrichment for intensively-farmed pigs
Source: PLoS One. 2019 Feb 22;14(2):e0212610. doi: 10.1371/journal.pone.0212610 (PMC6386313; doi:10.1371/journal.pone.0212610)
Supplement: S1 Fig — (DOCX) [file pone.0212610.s006.docx]

**S5 Fig.**

**Figure. Enrichment in 841 pens on 47 Dutch conventional pig farms in 2011.**

Our colleague, Herman Vermeer, kindly provided a Dutch dataset used for general welfare monitoring of growing-finishing pigs in 841 pens on 47 Dutch pig farms in 2011. Farms with evident participation in a welfare scheme (e.g. Better Life and organic farms) had been excluded as much as possible, as this survey concerned enrichment in conventional farming only. Participation of farmers was voluntary, hence there could be an overrepresentation of ‘better welfare’ farms in the dataset.

All 47 farms had partly-slatted floors, 31 farms had hopper feeders and 16 farms had feeding troughs. On average there were 12.8 pigs/pen provided with on average 0.89 m^2^/pig (average stocking density per farm ranged from 0.6 to1.3 m^2^/pig).

The results on the types of enrichment that were provided in the Netherlands in 2011 are shown in the Fig below.


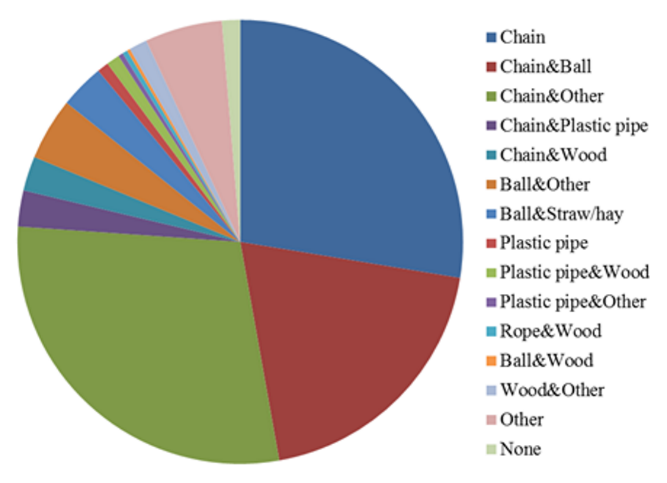


**Fig.** **Enrichment in 841 pens on 47 Dutch conventional pig farms in 2011.** (Data provided by H. Vermeer).

27.5% of pens had only a chain (label „Chain“ in Fig 1), 19.5% had a chain with a ball; 1.3% of pens had no enrichment at all (label ‚None‘).

81% of pens had a chain (with or without some other kind of enrichment), 27.6% had a Ball, 4.8% had a Plastic pipe, 5.5% had Wood.

Our impression is that compared to 2011 there is tendency for fewer bare chains, fewer balls and more plastic pipes being provided on conventional Dutch pig farms at present.
